# Semi-Continuous Flow Biocatalysis with Affinity Co-Immobilized Ketoreductase and Glucose Dehydrogenase

**DOI:** 10.3390/molecules25184278

**Published:** 2020-09-18

**Authors:** Michal Plž, Tatiana Petrovičová, Martin Rebroš

**Affiliations:** Institute of Biotechnology, Faculty of Chemical and Food Technology, Slovak University of Technology, Radlinského 9, 812 37 Bratislava, Slovakia; michal.plz@stuba.sk (M.P.); tatianapetrovicova@gmail.com (T.P.)

**Keywords:** ketoreductase, glucose dehydrogenase, immobilization, affinity, reactor

## Abstract

The co-immobilization of ketoreductase (KRED) and glucose dehydrogenase (GDH) on highly cross-linked agarose (sepharose) was studied. Immobilization of these two enzymes was performed via affinity interaction between His-tagged enzymes (six histidine residues on the N-terminus of the protein) and agarose matrix charged with nickel (Ni^2+^ ions). Immobilized enzymes were applied in a semicontinuous flow reactor to convert the model substrate; α-hydroxy ketone. A series of biotransformation reactions with a substrate conversion of >95% were performed. Immobilization reduced the requirement for cofactor (NADP+) and allowed the use of higher substrate concentration in comparison with free enzymes. The immobilized system was also tested on bulky ketones and a significant enhancement in comparison with free enzymes was achieved.

## 1. Introduction

Enzyme based biocatalyst plays an important role in organic synthesis and in the establishment of many chemical industries, e.g., fine chemicals, food or energy, textiles, agricultural, cosmeceutical, medicinal, and pharmaceutical industries [1].

The use of ketoreductases is well recognized for enantioselective reduction of prochiral ketones into stereo defined chiral alcohols [2,3]. Many wild strains of microbes can effectively reduce ketones, e.g., *Saccharomyces cerevisiae, Candida magnoliae* [4], *Sporobolomyces salmonicolor* [5], and *Pichia minuta* IAM 12215 [6]. Ketoreductases from wild strains are usually expressed in *E.coli* for effective carbonyl reduction, e.g., ketoreductase from *Pichia glucozyma* [7], and ketoreductase from *Hansenula polymorpha* [8]. Various types of ketoreductases can be implemented in the production of drugs, such as Ipatasertib (Roche) [9], Montelukast (Singulair^®^ by Merck, Sharp and Dohme) [10], Atorvastatin (Sortis^®^, Atorvalan^®^ or Lipitor^®^ by Pfizer) [11], and Sitaglipin (Merck, Sharp and Dohme) [12]. In an effective carbonyl reduction, there is a need for increased productivity and biocatalyst stability in terms of repeated use and process capacity. Thus, besides expressing ketoreductases in *E. coli*, enzyme immobilization can also be beneficial for the reduction process allowing the repeated and continuous use. Immobilization is a technique used for the physical or chemical fixation of a biocatalyst (cells/enzymes) and can be divided into three groups: (i) binding to a prefabricated support (carrier), (ii) entrapment in organic or inorganic polymer matrices, and (iii) cross-linking of enzyme molecules [13]. Ketoreductase was successfully immobilized via various techniques. Nagayama et al. immobilized commercially available ketoreductase on nonporous glass support to reduce 2-butanone to (*S*)-butanol [14]. Li et al. immobilized ketoreductase via covalent binding on EC-HFA resin (epoxide-functionalised support) for the production of (*R*)-1-(3,5-Bis(trifluoromethyl)phenyl)ethanol in flow reactor mode [15]. Ketoreductase can also be co-immobilized with auxiliary enzymes to enhance process efficacy, e.g., glucose dehydrogenase for efficient cofactor (NADPH/NADH) regeneration. Petrovičová and colleagues performed co-immobilization of ketoreductase and glucose dehydrogenase via entrapment into PVA particles [8].

The preparation of cross-linked enzyme aggregates can be also employed for successful enzyme immobilization [16]. Ning et al. reported the preparation of combi-CLEAs of ketoreductase and D-glucose dehydrogenase for the efficient conversion of 4-chloro-3-oxobutanoate [17]. However, preparation of cross-linked enzyme aggregates (CLEAs) was also employed for co- immobilization of different types of enzymes e.g., glucose oxidase and versatile peroxidase [18]; lipase, α- amylase and phospholipase A_2_ [19]; X-prolyl-dipeptidyl aminopeptidase and general aminopeptidase N [20]. CLEAs can achieve a high percentage of catalytic material per total mass of the immobilized biocatalyst, though a loss of enzymatic activity can arise from conformational restrictions, cross-linking, and blocked active sites [21]. Summarization of all co-immobilized systems goes beyond the scope of this paper, but Xu et. al. [16], Jia et. al. [22], and Kazenwadel et. al. [23] wrote excellent reviews in this field, which provide much better insights in the problematics of enzyme co-immobilization.

The harsh immobilization techniques mentioned above may lead to a significant decrease of enzymatic activity. Affinity binding between an enzyme and a material can overcome many of the challenges in other immobilization methods, especially possible decrease of activity caused by nonspecific reactions between enzyme and support. The binding usually occurs in an expected orientation based on the geometry of the affinity partners, lessening the possibility of steric blocking of the active site [24]. Enzymes tagged with affinity ligands (His-tag, Arg-tag, HAT-tag, maltose-binding protein, etc.) are less prone to inactivation during the immobilization process [25,26]. This type of immobilization can be reversible and can be employed on untreated materials with high loading efficiency. Fortunately, many enzymes have been produced with genetically encoded tags [27,28,29]. The His-tag, hexahistidine, is often applied for the purification of recombinant proteins. The binding affinity (K_d_) of single-His_6_ proteins for Ni-NTA modified surfaces has been estimated as ~1 µM [30]. So, this type of coordination bond can be categorized in moderate affinity within K_d_ range between 10^−4^ and 10^−7^ M. The typical values of *K_d_* for antibodies are in the nM (10^−9^ M) ranges. Despite a few drawbacks, such a relatively moderate affinity (*K_d_*~1 µM) [31], low specificity [32], and possible antagonistic effects on the activity of enzymes that contain divalent ions [33] His-tag can still be used for direct enzyme immobilization from crude cell extracts [34]. In principle, we cannot exclude the possibility of the affinity tag interfering with protein activity [35], although the relatively small size and charge of poly-His tag ensure that the protein is rarely affected.

A potential drawback when using IMAC might be the metal ion leakage from the resin, which can cause contamination of the final product. The leakage, however, can be minimized by charging the column with 70–90% of maximum metal ion capacity. By doing so, the leaching metal ions will be recaptured by the unloaded resin at the bottom of the column [36].

Another way to improve the biocatalytic process is the use of a flow reactor with immobilized enzymes [37], where the larger productivity, mixing efficiency, continuous production, and better product recovery can be achieved [38].

There are many examples of successful use of flow reactors for many types of reactions, e.g., hydrolysis and formation of esters [39,40] formation of C-C bonds by transketolases [41], preparation of monosaccharides [42] and oligosaccharides [43]. Flow reactors were also employed for reactions with co-immobilized ketoreductase and auxiliary enzyme, which perform cofactor regeneration within the same reactor [44]. There are several types of reported flow reactors with immobilized ketoreductase, e.g., for reduction of bulky ketones [45], oxidation of *n*-hexanol [46], and preparation of chiral alcohols [15].

Despite the abovementioned advantages of flow reactors, the major challenge lies in the immobilization of isolated enzymes that often leads to a significant decrease in enzymatic activity. Hence, the enzymes with His-tags can be applied for construction of semicontinuous flow reactors. This approach combines the nonaggressive immobilization method followed by the use of reactor, which offers the benefits of enhanced productivity and process control. For this reason, this work is focused on performation of a direct affinity co-immobilization of enzymes from crude enzyme extracts followed by the application of semiflow reactor for repeated biotransformations, to minimize the addition of cofactors, and increase the substrate concentrations in biocatalysis processes.

## 2. Results and Discussion

### 2.1. Ketoreductase and Glucose Dehydrogenase Expression

The ketoreductase gene from *Hansenula polymorpha* [47] and the gene for glucose dehydrogenase (EC. 1.1.1.47) from *Bacillus megaterium* were selected as reported before [8]. Enzymes were expressed in *E. coli* BL21(DE3) with a combined *lac* and T7 promoter. The presence of the enzymes in cells was controlled via SDS-PAGE (Figure S1; Supplementary Material). The activities of the expressed enzymes were measured spectrophotometrically by cofactor utilisation for each enzyme crude cell extract. The initial specific activity of the enzymes produced (ketoreductase and glucose dehydrogenase) was measured with fermentation samples diluted to the desired OD_600_ of 10. The initial activity for ketoreductase and glucose dehydrogenase was 8.46 U and 34.7 U, respectively.

### 2.2. Biotransformation with Free Enzymes

**Figure 1:**
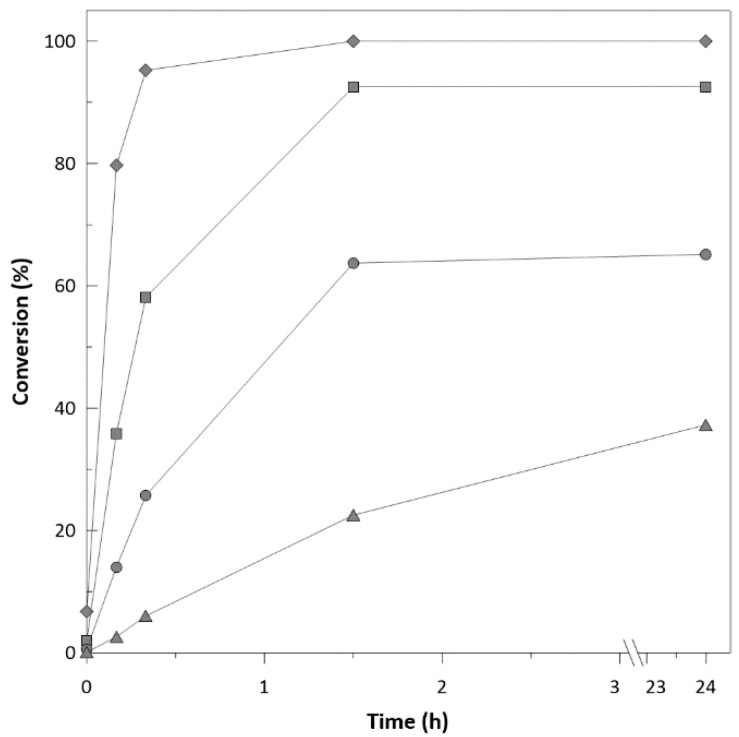
Achieved substrate conversion with different initial substrate concentrations (ethyl-2-methyl-acetoacetate: ♦ 35 mM; ■ 70 mM; ● 100 mM; ▲ 130 mM).

To characterise the performance of enzymes during biotransformation, reactions with free enzymes and substrate ranging in concentrations from 35 mM to 130 mM were performed. At initial substrate concentrations higher than 70 mM (Figure 1), reactions were significantly inhibited and reached a substrate conversion after 24 h of only 65.5% and 37.7 % for 100 mM and 130 mM, respectively. Decrease of the enzyme initial activity was also observed (Figure S5; Supplementary Material) from 2.56 to 0.09 U.mg^−1^_protein_, respectively.

**Figure 2:**
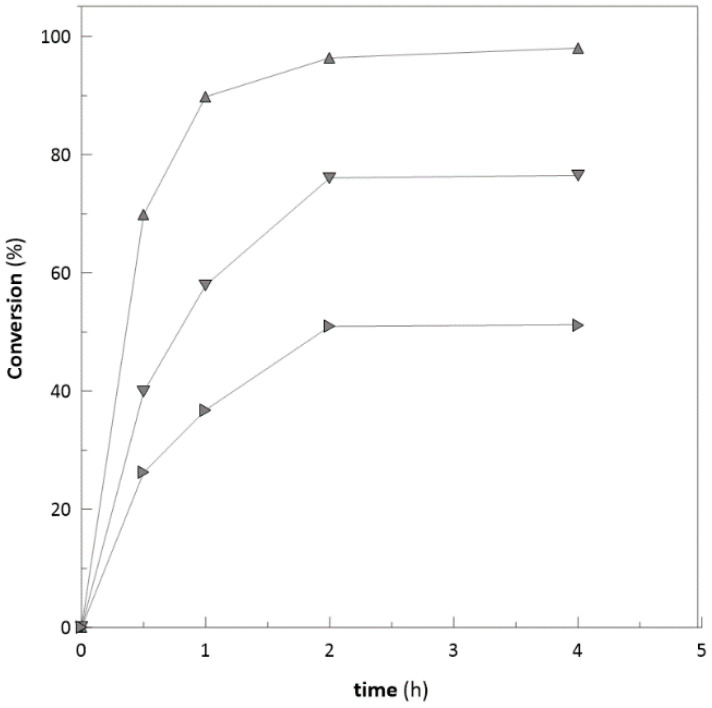
Achieved substrate conversion with different initial substrate concentrations (4-phenyl-2-butanone: ▲ 20 mM; ▼ 50 mM; ► 100 mM).

**Figure 3:**
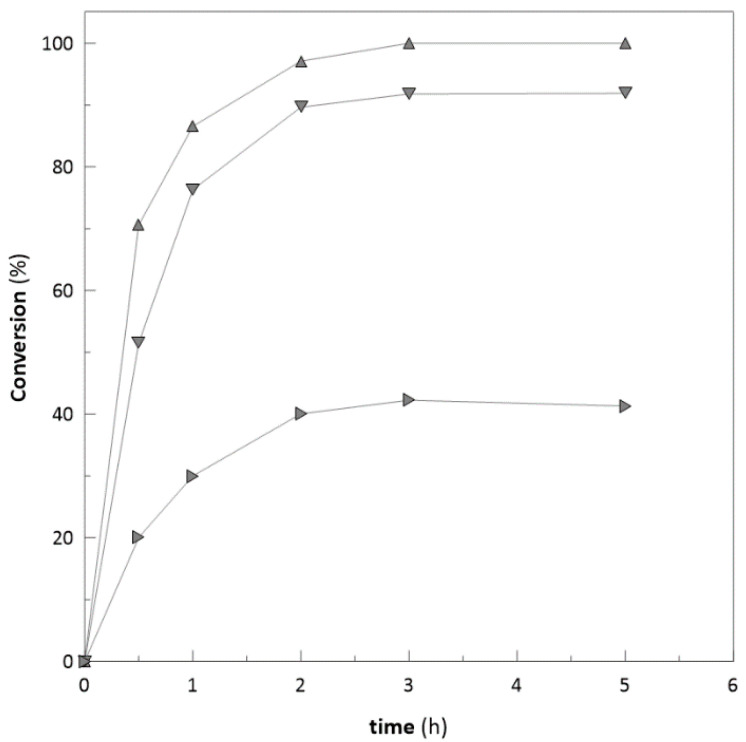
Achieved substrate conversion with different initial substrate concentrations (3′-hydroxyacetophenone: ▲ 20 mM; ▼ 50 mM; ► 100 mM).

To characterise the enzyme robustness, the ketoreductase was also used for reduction of some bulky ketones, e.g., 4-phenyl-2-butanone and 3′-hydroxyacetophenone with substrate concentration of 25, 50, and 100 mM, respectively. In the case of 4-phenyl-2-butanone with concentration of 20 mM, the complete conversion was achieved. However, with concentrations of 50 and 100 mM (Figure 2), the reactions were inhibited at the 4th hour of bioconversion and achieved only 80 and 53.2% conversion after 24 h (data not shown). Similar trend was observed in the case of 3′-hydroxyacetophenone. Substrate concentrations of 20 and 50 mM achieved almost complete conversions (98 and 80%). However, the concentration of 100 mM (Figure 3) was inhibited at the 5th hour and achieved only 40.2% after 24 h of biotransformation (data not shown). Ethyl-2-methylacetoacetate and similar types of substrates are often used at low concentrations (25–50 mM) due to their potential to inhibit enzymes [48].

### 2.3. Ketoreductase and Glucose Dehydrogenase Co-immobilization

Two initial strategies for enzyme load were tested in optimisation experiments: separate zonal load of ketoreductase (KRED) and glucose dehydrogenase (GDH) crude cell extracts, and homogenous load of both mixed crude cell extracts (Figure S2; Supplementary Material). Only a slight difference was observed between these two loading methods in terms of biocatalyst initial activity and conversion (16.5 U/mL resin for zonal load and 18 U/mL resin for homogenous load with the same amounts of crude extracts loaded and the same biotransformation conditions; 70 mM of ethyl-2-methyl-acetoacetate in both cases—Figure S3; Supplementary Material). For this reason, co-immobilization of KRED and GDH was investigated further using the homogenous load method.

**Figure 4:**
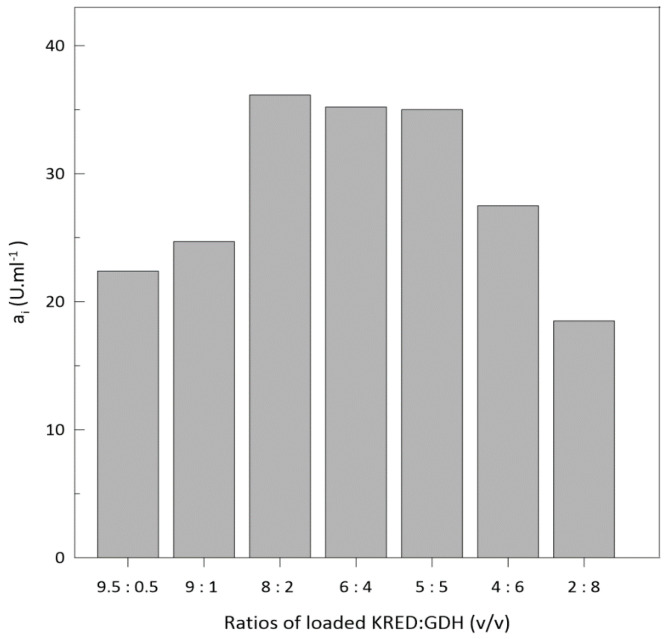
Activity of different enzymes’ loading ratio during immobilization.

The loading of crude enzyme extract was carried out to keep the immobilization process simple and cheap (Figure S4; Supplementary Material). Both crude enzyme extracts were prepared with identical disruption of biomass diluted to an OD_600_ of 60. To find the proper ratio of enzymes, both crude enzyme extracts were mixed at different ratios and then loaded onto the column. Seven ratios of mixed crude cell extracts were tested (Figure 4) to identify the main enzyme (ketoreductase) and cofactor regenerating enzyme (glucose dehydrogenase) amounts.

The ratio with the highest activity of KRED/GDH was 8:2 (*v*/*v*). During the immobilization process, loaded enzymes are competing for the binding capacity of the matrix. Ketoreductase was used in excess because it has lower stability during biotransformations [49] and is a rate-limiting enzyme. In the literature, the authors used different ratios of KRED and GDH for performing biotransformation reactions, e.g., 8:1 [50], 1:1 [49], 5:1.7, and 5:6.9 [8]. We conclude that loading ratios heavily depend on initial enzyme activity, the immobilization method used, and the potential loss of activity during the Immobilization process itself. Characterization pH and temperature (Figure S5; Supplementary Material) was also performed. The highest activity was achieved at pH 6.5 and temperature 37 °C. These results are similar when compared to the free form of ketoreductase [8].

### 2.4. Optimisation of Cofactor (NADP^+^) Concentration

A very important factor in most of the cofactor-dependent redox processes is the cofactor concentration. Due to its price (100 mg ~ 78 €; www.sigmaaldrich.com), it is impossible to add it to a reaction in an equimolar ratio. Therefore, the cofactor regeneration system is usually applied, and the cofactor addition needs to be optimised in the reaction set-up. A cofactor is often used in amounts up to 1% (*w*/*w*) relative to the substrate [51].

**Figure 5:**
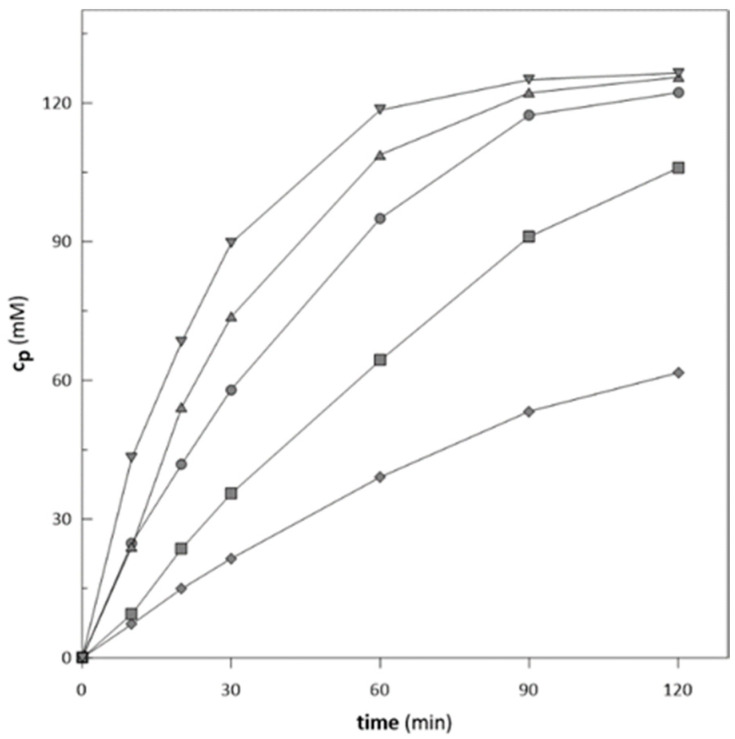
Biotransformations of ethyl-2-methyl acetoacetate at different cofactor concentration (♦ 10 µM; ■ 25 µM; ● 50 µM; ▲ 125 µM; ▼ 250 µM) with immobilized enzymes.

**Figure 6:**
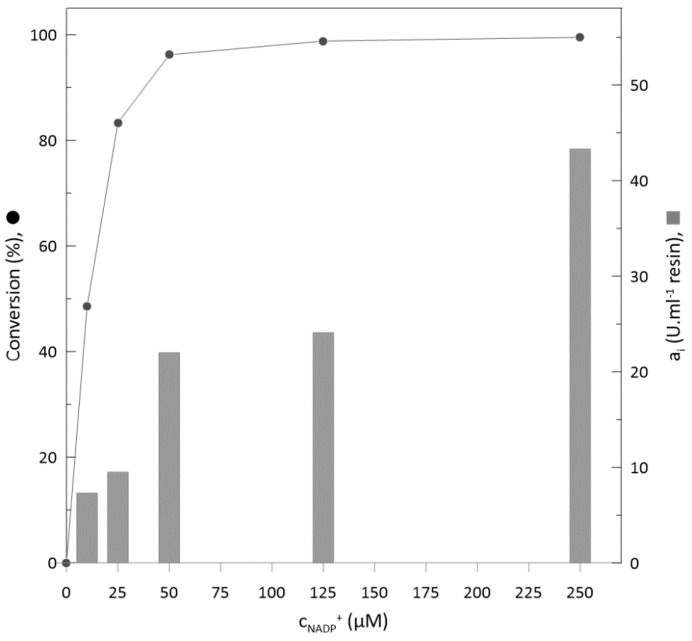
Substrate conversions and initial activity of immobilised enzymes with different cofactor concentrations.

Biotransformations with an enzyme load of 8:2 (ketoreductase: glucose dehydrogenase; *v*/*v*) and substrate concentration of 130 mM were applied for five different cofactor concentrations ranging from 10 µM to 250 µM. We evaluated the initial activity and conversions reached (Figure 5 and Figure 6) in the biotransformation process. With increasing concentration of NADP^+^, the initial activity significantly increases (Figure 5). However, considering the final conversion at 120 min of the biotransformation, it is obvious that 50 µM of NADP^+^ is a sufficient amount to reach complete substrate conversion at the same time as for 125 µM and 250 µM, respectively (Figure 5 and Figure 6). On the other hand, the reaction duration can be crucial in some cases. For example, during cascade reactions, it is important to hold the reaction time of the first reaction at a steady pace, and an increase in reaction time on the behalf of lower cofactor consumption is not the best optimization option [52].

As reported before, with the cofactor regenerating system (e.g., glucose dehydrogenase) the amount of cofactor can be lowered to catalytical amount, i.e., 1% (*w*/*w*) relative to the substrate [51] (e.g., 100 mM substrate + 1 mM of NADP^+^). As reported in this publication, the cofactor was lowered to 0.03% (relative to the substrate). In comparison with other studies, this is the lowest amount of cofactor that has been reported (0.2% [45]; 2.85% [8], 0.1% [51], and 0.2% [47]), which may significantly decrease the biocatalysis cost in upscale processes of this system.

### 2.5. Substrate Concentration

The critical point in biocatalysis applications is often the initial concentration of the substrate combined with the conversion reached. This is also what determines its application in industry [53]. Therefore, a series of biotransformations with substrate concentrations ranging from 35 mM to 700 mM were performed. When working with ketoreductase, the main issue is the potential inhibitory effect of the substrate on enzyme activity, or its low solubility in aqueous solutions. In our case, the difficulties with substrate solubility became apparent above a concentration of 700 mM.

**Figure 7:**
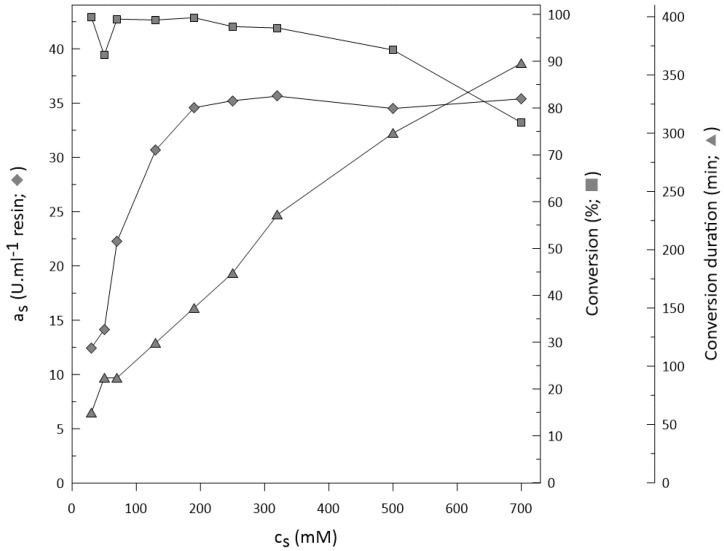
Initial activity, substrate conversion, and time needed for completion of each conversion with different substrate concentrations.

We can assume that with a substrate concentration above 200 mM, sufficient conversion (>93%) and specific activity (Figure 7) can be achieved. The concentrations of substrate used for ketoreductase, as described in the literature, is rarely above 100 mM (97 mM [47], 50 mM [45], and 35 mM [8]). Hence, the studied method of ketoreductase and glucose dehydrogenase co-immobilization is a beneficial step for biocatalytic application of ketoreductase, making it an interesting choice for industrial applications. The biotransformation process becomes relevant in drug manufacturing when the concentration of substrate is above 100 g/L, e.g., 100 g/L in the synthesis of Montelukast^®^ [10], and 160 g/L for Atorvastatin^®^ [11], both prepared by ketoreductase. In this publication, we achieved 92.4% conversion with a substrate concentration of 72 g/L (500 mM) and 76.9% conversion with a substrate concentration of 100 g/L (700 mM).

In comparison with free enzymes, the immobilised biocatalyst was stable in a broad range of substrate concentration (from 70 mM to 700 mM). In the reaction with free 0.97 mg of KRED in a total volume of 1 mL, the observed inhibition concentration was above 100 mM. On the other hand, when working with immobilised enzymes, the theoretical value of ketoreductase in 1 mL column is 10 mg, and this amount was used for the reaction in a total volume of 10 mL. The mass ratio used of free and immobilised enzyme was almost equal. Therefore, the immobilization of enzymes plays a protective role in protein stability. For all enzymes, the potential to be immobilised and used in heterogeneous form brings important industrial and environmental advantages.

#### Application on Bulky Ketones

**Figure 8:**
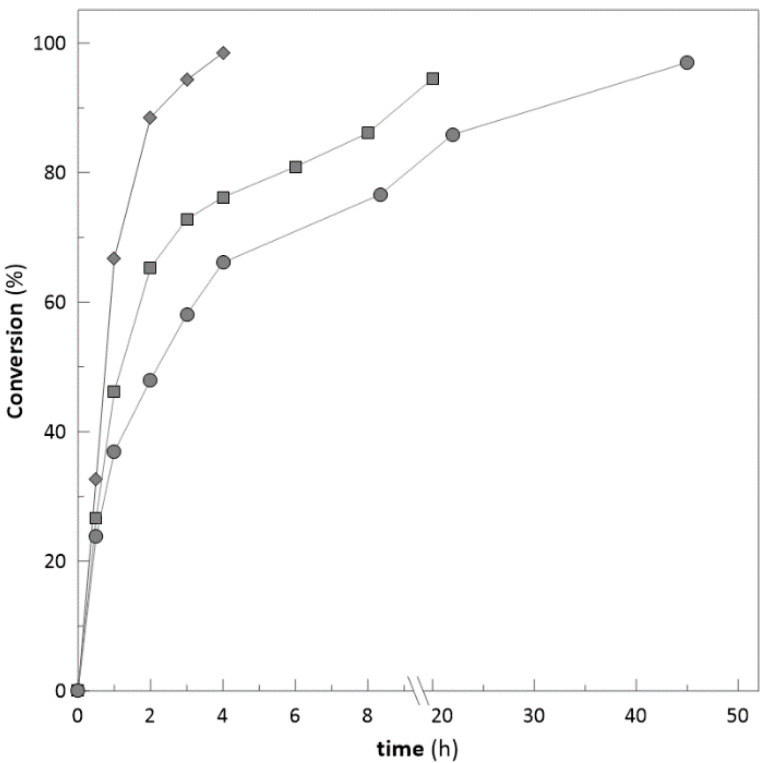
Achieved substrate conversion with different concentration of 4-phenyl-2-butanone (♦ 20 mM; ■ 50 mM; ● 100 mM).

**Figure 9:**
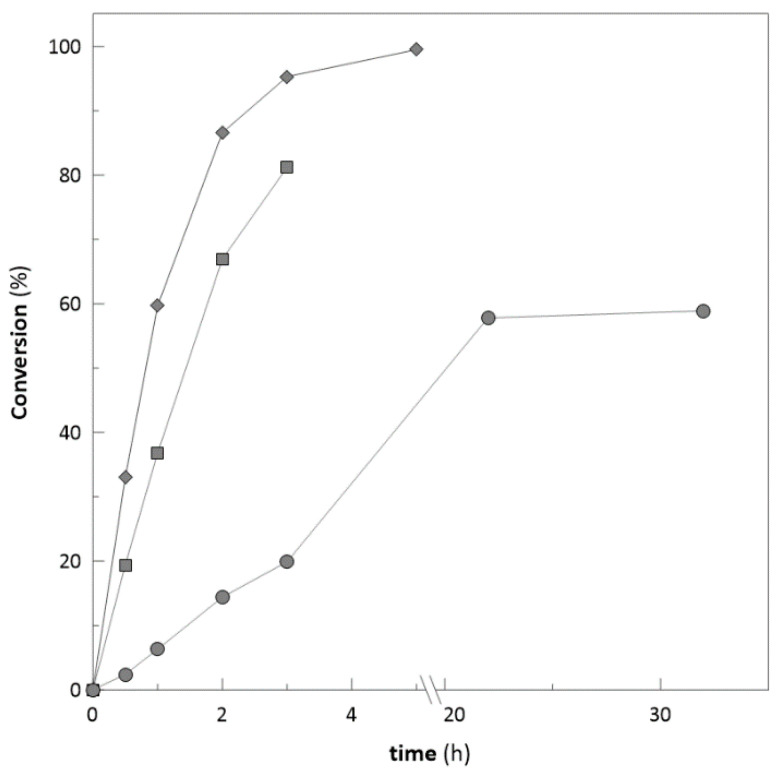
Achieved substrate conversion with different concentration of 3′-hydroxyacetophenone (♦ 20 mM; ■ 50 mM; ● 100 mM).

After successful application on the model substrate (ethyl-2-methyl acetoacetate), the immobilized biocatalyst was used for biotransformations of bulky ketones, e.g., 4-phenyl-2-butanone and 3′-hydroxyacetophenone to test the robustness of prepared biocatalyst. Reactions were carried out with immobilized biocatalyst and compared with free enzymes (Figure 2 and Figure 3) respectively in presence of DMSO up to 5% (*v*/*v*) and with a substrate concentration ranging from 20 to 100 mM. During the reactions with 4-phenyl-2-butanone and 3′-hydroxyacetophenone with substrate concentration of 20 mM, no significant difference was observed when comparing free and immobilized biocatalyst. However, with an increase of the substrate concentration to 50 and 100 mM (4-phenyl-2-butanone) immobilized biocatalyst achieved almost the complete conversion of substrate (95.5% and 97%) (Figure 8), whilst free enzymes achieved only 80 and 53.2% (Figure 2). The improvement of conversions was also observed for 3′-hydroxyacetophenone (Figure 9) with concentrations 50 and 100 mM. With substrate concentration of 50 mM the immobilized system achieved a higher degree of conversion 98.5% (92%—free enzyme-Figure 3). On the other side, with a substrate concentration of 100 mM, immobilized biocatalyst achieved conversion only 60% (40%—free enzyme-Figure 3).

From achieved results it can be assumed that use of immobilized biocatalyst in semicontinuous flow mode can be employed also for the reduction of bulky ketones. When compared with the same amount of free enzymes, at higher substrate concentration the higher substrate conversion can be achieved. Although only two substrates from bulky ketones were tested, the similar trend can be expected in the use of other substrates. As discussed before (Section 2.5), the immobilization of enzymes plays a protective role in protein stability [44].

### 2.6. Repeated Biotransformations

**Figure 10:**
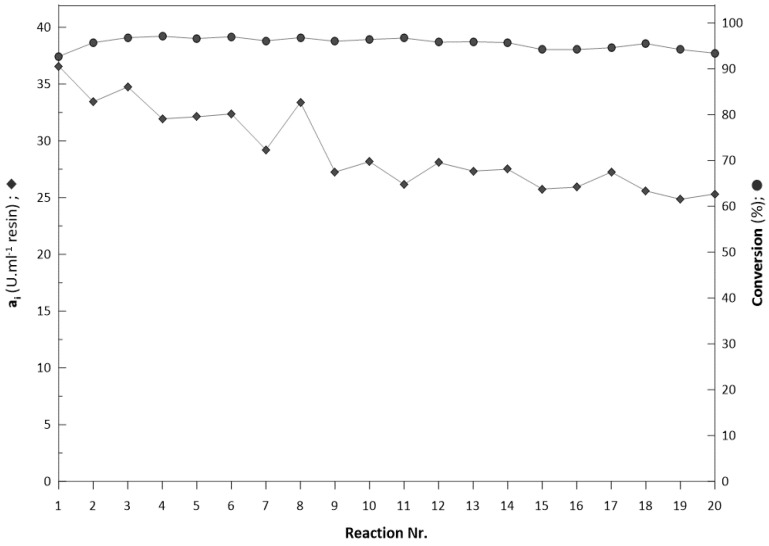
Repeated biotransformation with substrate concentration of 130 mM.

**Figure 11:**
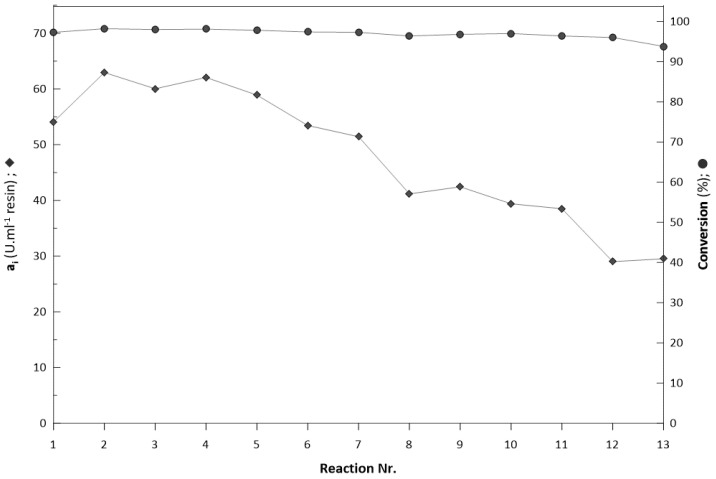
Repeated biotransformation with substrate concentration of 190 mM.

Two series of repeated batch biotransformations in a semicontinuous flow reactor with different substrate concentrations of 130 mM (Figure 10) and 190 mM (Figure 11) were performed. To test the operational stability of prepared biocatalyst, both series were performed at the same conditions in nonstop mode. In the first set of reactions (Figure 10), twenty biotransformations were performed. The biotransformations were set to achieve a minimum substrate conversion of 95%. The initial activity of 35 U/mL was achieved during the 1st batch biotransformation. In comparison with the reaction of the free enzyme under the same conditions, the reaction occurred very slowly and stopped at a substrate conversion of 37.7% (Figure 1). After each reaction, the column (5 mL) was washed with approximate 100 mL of potassium phosphate buffer (pH 6.5) to remove excessive traces of product before the following reaction to prevent a likely inhibitory effect.

A 32% decrease of initial immobilised biocatalyst activity (a_i_) occurred (Figure 10, Figure S7; Supplementary Material). The significant decrease in activity occurred during the initial nine reactions, where activity decreased from 36 U/mL to 27 U/mL and remained stable until the 20th reaction. In all reactions, the elution of proteins from the column was not detected. Similar nonlinear decreases in activity were reported previously in entrapment studies with enzymes immobilised in LentiKats^®^ [8,54], which evokes the stabilisation of immobilised enzymes activities in reactions conditions. In the second set of reactions with an initial substrate concentration 190 mM (Figure 11, Figure S8; Supplementary Material), thirteen repeated biotransformations were performed with substrate conversion > 95%. The decrease of initial activity (a_i_) was more significant, since 70% of initial activity was lost in 13 reactions.

This may be caused by the effect of the content of non-natural chemicals on enzyme stability, as reported by [55], given that enzymes are often applied in concentrated systems. Long and linear chain alcohols can also play an important role in the disturbance of hydrophobic interactions as well as the hydrogen-bonding of proteins [56].

By both processes, at lower (130 mM) (Figure 10) and higher (190 mM) substrate concentration (Figure 11), a total of 35 g of secondary alcohol was produced (Section 3.6.2).

In comparison with recent research, this is the first to describe direct immobilization of ketoreductase from crude cell extracts and consecutive batch reactions in a flow reactor. Dall’ Oglio and colleagues describes a robust two-enzyme system composed of immobilised ketoreductase and glucose dehydrogenase on aldehyde activated agarose used in a flow reactor for weeks, even in the presence of DMSO up to 20% (*v*/*v*) [45].

Immobilization on aldehyde agarose relies on the reaction between nonprotonated ε-amino groups of surface lysines and aldehyde groups on the support. Alkaline conditions (pH ≥ 10) are required to ensure that the ε-amino groups are not protonated. However, the glucose dehydrogenase lost > 50% of the initial activity after 1 h of incubation at pH 10. Hence, a stabilising agent must be added (i.e., glycerol or PEG600). On the other hand, ketoreductase retained 100% of its initial activity over 3 h of incubation at pH 10. Reaction buffers in flow mode contained various ketones with a concentration of 3 mM. The conversions achieved on the 1st and 15th day were stable, and ranged from 62% to 97%, depending on the ketone used [45].

Other immobilizations of KRED were reported by Petrovičová et al., when they co-immobilised ketoreductase and glucose dehydrogenase in PVA particles (LentiKats^®^) by entrapment. They also performed twenty consecutives biotransformations with a small loss of initial enzyme activity and conversions above 95%. However, only concentrations of substrate up to 35 mM were used [8].

Peschke and colleagues [34] constructed a compartmentalised microfluidic packed-bed reactor loaded with enzyme-functionalised magnetic beads (MB). The beads were functionalised with cobalt (Co^2+^), for selective binding of the His-tagged enzymes: (*R*)-selective alcohol dehydrogenase and glucose dehydrogenase via formation of a coordination (affinity) bond. MB-Co^2+^ were incubated with an excess of the purified enzymes and then loaded into the individual compartments of a polymethylmethacrylate chip comprising of four linear flow channels that could be connected in parallel. The reactor showed a 5-nitrononane-2,8-dione (NDK) conversion of 98%, and a space-time yield of 131 g/L per day.

In comparison with the processes mentioned above, the process developed in this work does not require further enzyme pre-isolation and prepurification, and it does not require an immobilization matrix, which can be in some cases very difficult and expensive process. The commercially available matrix, Ni Sepharose^®^ High Performance (Sigma Aldrich, St. Louis, Missouri, USA), with the primary function of His-tag protein isolation, can be directly used for enzyme immobilization from crude cell extract, which eliminates the additional immobilization cost. Hence, our process is very simple, easy to perform, and has the potential for scale-up.

On the other side, there is a lot of examples of other enzymes immobilised via coordination (affinity) bond, e.g., adenylate cyclase (AC) on agarose particles [57], lipase on magnetic nanoparticles (Ni-NTA-MNPs) [58], and O-Acetylserine sulfhydrylase (OASS) on iron oxide magnetic nanoparticles [59]. However, as reported in these studies, many of these techniques require complete isolation and purification of the enzymes, and complete synthesis of a novel immobilization matrix. Therefore, the main advantage of our immobilization technique, when compared to others, is its simplicity to perform.

### 2.7. Storage of the Immobilised Biocatalyst

The immobilised biocatalyst was stored in storage solution at 4 °C. Immobilized biocatalyst remained 100% of its activity for the first 50 days of storage, and then the activity started to decrease to 50% during 100 days, and then remained stable. Similar phenomenon of continual activity decrease of to 50% at the same storage conditions was observed after 20 days in the case of free enzyme (Figure S9; Supplementary Material).

## 3. Materials and Methods

### 3.1. Chemicals and Media

Bacteria were inoculated in Luria-Bertani (LB) medium containing (per litre of distilled water): 5 g yeast extract, 10 g NaCl, 10 g tryptone, and 30 mg kanamycin; plates contained an additional 20 g of agar. Production medium included the following ingredients (per litre): 30 g glycerol, 10 g tryptone, 2.8 g NaH_2_PO_4_, 3.87 g KCl, 4 g citric acid, 5 g (NH_4_)_2_SO_4_, 2 g MgSO_4_, 30 mg kanamycin, and 10 mL of trace element solution. Trace element solution contained (per litre): 10 g citric acid, 0.34 g CaCl_2_.2H_2_O, 0.246 g ZnSO_4_.7H_2_O, 0.152 g MnSO_4_.H_2_O, 0.05 g CuSO_4_.5H_2_O, 0.0427 g CoSO_4_.7H_2_O, 0.967 g FeCl_3_.6H_2_O, 0.003 g H_3_BO_3_, and 0.0024 g Na_2_(MoO_4_).2H_2_O. Feeding medium (for fed-batch fermentation) contained (per litre): 630 g glycerol, 114.4 g NaH_2_PO_4_, and 170 g MgSO_4_.7H_2_O.

Kanamycin was purchased from Gibco^®^ (Life Technologies, Glasgow, UK), ethyl-2-methylacetoacetate was purchased from Sigma-Aldrich (St. Louis, MO, USA), and NADP^+^ and NADPH were purchased from Prozomix (Haltwhistle, UK). Inductor isopropyl β-D-thiogalactoside (IPTG) was purchased from Thermoscientific (Waltham, MA, USA). All other chemicals were of analytical grade and commercially available.

### 3.2. Cloning

Codon-optimised genes encoding ketoreductase from *Hansenula polymorpha* [47] and glucose hydrogenase from *Bacillus megaterium* were obtained from Generay Biotech Co., Ltd. (Shanghai, China). Genes were inserted into a pET28b expression vector, and these plasmids were transformed into *Escherichia coli* BL21(DE3) competent cells.

### 3.3. Preparation of Ketoreductase and Glucose Dehydrogenase

An inoculum of *E. coli* BL21(DE3) expressing ketoreductase or glucose dehydrogenase was prepared from a single colony. Tubes containing 3 mL of LB medium were incubated overnight at 37 °C, shaking at 200 rpm. After approximately 12 h, a 500 mL flask containing 100 mL of medium was inoculated with 1% (*v*/*v*) inoculation culture and cultivated at 37 °C, shaking at 200 rpm.

After reaching an optical density at 600 nm (OD_600_) of 0.5–0.6 (about 3 h), the culture was used as the inoculum for the fermenters. Fed-batch fermentation was performed in a 3 L laboratory fermenter (BioFlo^®^ 115, New Brunswick, NJ, USA) with 2 L of production medium at 30 °C, shaking at 200 rpm (agitation was later maintained at an oxygen saturation of 30% by increasing agitation from 200 rpm to 1200 rpm), and pH 7 (buffered with 24 % (*v*/*v*) ammonia solution and 3.1 M phosphoric acid solution). An inductor (IPTG-0.1 mM) was added during the exponential stage of growth (range of optical density values OD_600_ = 50–70). After reaching an OD_600_ above 135 (with sufficient enzyme activity of ketoreductase and glucose dehydrogenase), cells from the fermenter were harvested via centrifugation (12,100× *g*, 30 min, 4 °C). The sediment was subsequently resuspended in 0.1 M potassium phosphate buffer (ketoreductase: pH 6.5; glucose dehydrogenase: pH 7) to a final OD_600 nm_ of 60. The cell suspension was disrupted in a continuous cell disruptor (CF Range, Constant Systems Ltd., Daventry, UK) in two cycles at 4 °C and 20 kPSI. After the disruption, the cell debris was removed by centrifugation (12,100× *g*, 30 min, 4 °C), and supernatants were applied for immobilisations.

### 3.4. Enzyme Assays

The ketoreductase assay (1 mL) contained 0.1 M potassium phosphate buffer (pH 6.5), 35 mM ethyl 2-methylacetoacetate, and 5 µL of ketoreductase cell lysate (diluted to OD_600nm_ = 10). The reaction was initiated with 0.5 mM NADPH, and its decrease was measured spectrophotometrically at a wavelength of 340 nm. The glucose dehydrogenase assay (1 mL) contained 0.1 M potassium phosphate buffer (pH 7), 100 mM glucose, and 5 µL of GDH cell lysate (diluted to OD_600nm_ = 10). The reaction was initiated with 0.5 mM NADP^+^. One unit of enzyme activity was defined as the amount of ketoreductase required to catalyse the oxidation of 1 µmol NADPH/min or the amount of glucose dehydrogenase required to catalyse the reduction of 1 µmol NADP^+^/min at pH 6.5 (ketoreductase) or 7 (glucose dehydrogenase) at 25 °C.

### 3.5. Co-Immobilisation

**Scheme 1:**
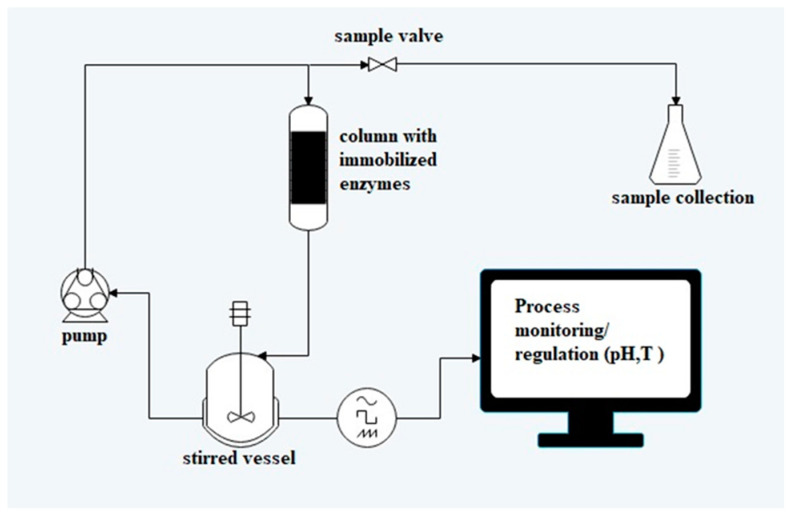
Reactor configuration for biotransformation.

The enzymes were immobilised on a 5 mL (or 1 mL) HisTrap^TM^ High-Performance column (GE Healthcare, Chicago, IL, USA). The column contained Ni^2+^ ions attached to sepharose (highly cross-linked agarose). Crude cell extracts (40 mL ketoreductase and 10 mL glucose dehydrogenase, in the case of 1 mL column the volumes were 5 times lower) were filtered (ADVANTEC^®^, Toyo Roshi Kaisha, Ltd., Japan; 0.45 µm) and loaded onto a 5 mL column equilibrated with binding buffer (pH 5.8, 300 mM sodium phosphate, 300 mM NaCl, and 10 mM imidazole). Nonbinding proteins were washed out with 25% (*v*/*v*) of elution buffer (pH 7.4, 300 mM sodium phosphate, 300 mM NaCl, and 500 mM imidazole) using an ÄKTA purifier system (GE Healthcare, Chicago, IL, USA). The presence of proteins in eluted fractions was determined by SDS-PAGE electrophoresis (Mini PROTEAN^®^ Tetra Cell, Bio-Rad, Hercules, CA, USA). The column with co-immobilised enzymes was then used for repeated biotransformations in a flow reactor in semicontinuous mode (Scheme 1). From previous purification experiments it was determined that 10 mL (8:2; KRED: GDH; *v*/*v*) of mixed crude cell extracts contains 11.48 mg of proteins (8.8 mg KRED and 2.68 mg of GDH). Hence, it can be assumed that the same amount of proteins can be found in 1 mL column and 57.4 mg in 5 mL column. The activity was calculated for 1 mL of resin with immobilised biocatalyst.

#### Optimization of Enzyme Loading into Column

**Scheme 2:**
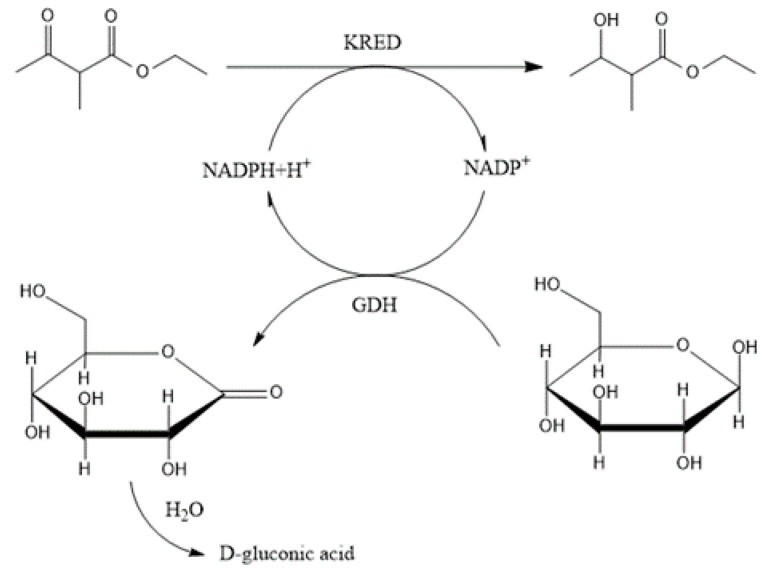
Reaction scheme of ketone reduction (ethyl-2-methyl-acetoacetate).

Crude cell extracts of ketoreductase and glucose dehydrogenase with OD_600_~60 were used in different volumetric ratios for the preparation of immobilised biocatalyst (1 mL column). The volumetric ratios were as follows (*v*/*v*): 9.5:0.5; 9:1; 8:2; 6:4; 5:5; 4:6; 2:8). Ethyl 2-methylacetoacetate was used as a β-ketoester substrate for the KRED biotransformations. The GDH regeneration system (Scheme 2) was used to regenerate NADP^+^. The reaction mixture contained 0.1 M potassium phosphate buffer (pH 6.5), 190 mM of ethyl-2-methyl acetoacetate, 300 mM and 250 µM NADP^+^. The reaction mixture recirculates through a column reactor according to the Scheme 1. Reactions were carried out in a tempered reactor in a total volume of 10 mL at 37 °C with mixing (250 rpm).

### 3.6. Biotransformations with Immobilised Enzymes

#### 3.6.1. Substrate 1: ethyl-2-methylacetoacetate

Ethyl 2-methylacetoacetate was used as a β-keto ester substrate for the KRED biotransformations. The GDH regeneration system (Scheme 2) was used to regenerate NADP^+^. The reaction mixture contained 0.1 M potassium phosphate buffer (pH 6.5), 130 mM (190 mM in the second set of reactions) of ethyl-2-methyl acetoacetate, 200 mM glucose (300 mM in the second set of reactions), and 250 µM NADP^+^. The reaction mixture recirculates through a column reactor according to the Scheme 1. Reactions were carried out in a tempered reactor in a total volume of 100 mL at 37 °C with mixing (250 rpm). In the repeated batch biotransformations, the column reactor was washed three times with 0.1 M potassium buffer (pH = 6.5) after each biotransformation. Initial activity (a_i_) was determined according to the biotransformation of substrate (1) and was related on the 1 mL of resin containing immobilised biocatalyst.

(1) $$a_{i\;}=\frac{\Delta n_{P}}{\Delta t.\;\;V_{resin}};for\;\Delta t=30\;min;\;\left[U/ml_{resin}\right]$$

#### 3.6.2. Substrates 2; 3: 4-phenyl-2-butanone; 3′-hydroxyacetophenone

4-Phenyl-2-butanone and 3′-hydroxyacetophenone were used as bulky ketones for the KRED biotransformations. The GDH regeneration system (Scheme 2) was used to regenerate NADP^+^. The reaction mixture contained 0.1 M potassium phosphate buffer (pH 6.5), 20 mM (50 and 100 mM) of 4-phenyl-2-butanone or 3′-hydroxyacetophenone, 100 mM glucose (200 and 400 mM), and 250 µM NADP^+^. The reaction mixture recirculates through a column reactor (1 mL) according to the Scheme 1. Reactions were carried out in a tempered reactor in a total volume of 10 mL at 37 °C with mixing (250 rpm).

### 3.7. Biotransformations with Free Enzymes

#### 3.7.1. Substrate 1: ethyl-2-methylacetoacetate

The reaction mixture contained (per litre): 0.1 M potassium phosphate buffer (pH 6.5), 35 (70, 100, 130) mM ethyl 2-methyl acetoacetate, 100 (200, 280, 360) mM glucose, 1 mM NADP^+^, 0.971 mg of isolated KRED, and 0.038 mg of isolated GDH. The amount of buffer added depended on the volume of the enzyme used. Reactions were performed in Eppendorf tubes (T = 37 °C, 550 rpm) in a total volume of 1 mL.

#### 3.7.2. Substrates 2; 3: 4-phenyl-2-butanone; 3′-hydroxyacetophenone

The reaction mixture contained (per litre): 0.1 M potassium phosphate buffer (pH 6.5), 20 (50, 100) mM 4-phenyl-2-butanone or 3′-hydroxyacetophenone, 100 (200, 400) mM glucose, 0,25 mM NADP^+^, 1,1 mg of isolated KRED, and 1,1 mg of isolated GDH. The amount of buffer added depended on the volume of the enzyme used. Reactions were performed in Eppendorf tubes (T = 37 °C, 600 rpm) in a total volume of 1 mL.

### 3.8. Analytics

#### 3.8.1. Substrate 1: ethyl-2-methylacetoacetate

During the biotransformations, 100 µL of sample was taken at regular intervals, and 0.3 mL of ethyl acetate was added to extract both the substrate and product. After vortexing, the sample was centrifuged (13,300× *g*, 1 min) to facilitate the phase separation. The organic phase was transferred to a glass vial for analysis. The conversion of keto ester to hydroxy ester was monitored by gas chromatography with flame ionization detector (GC-FID, Agilent 6890N, Santa Clara, CA, USA) with DB-5 capillary column (Agilent J&W, 30 m × 0.25 mm × 0.25 µm) and hydrogen (H_2_) as the carrier gas with a flow of 1 mL/min. The injection volume was 1 µL with a 50:1 split. The oven temperature was initially 90 °C for 5.5 min, then increased to 280 °C with a gradient of 30 °C/min, and was held for 3 min.

The conversion was evaluated as the peak area of the alcohol product (peak area of ketone substrate + peak area of alcohol product) × 100. One unit of ketoreductase was the amount of enzyme that catalysed the production of 1 µmol/min of hydroxy ester (Scheme 2). The initial specific activity of free ketoreductase was defined as µmol of hydroxy ester produced/min by 1 mg of ketoreductase. The specific activity of the immobilised enzyme was defined as 1 µmol/min produced by 1 mL of resin with immobilised enzymes.

#### 3.8.2. Substrates 2; 3: 4-phenyl-2-butanone; 3′-hydroxyacetophenone

Samples were taken and prepared as in the Section 3.7.1 and the conversion was calculated in the same way.

The conversion of 4-phenyl-2-butanone was monitored by chromatography with flame ionization detector (GC-FID, Agilent 7890B, Santa Clara, CA, USA) with CP-Chirasil-Dex CB capillary column (Agilent J&W, 25 m × 0.25 mm × 0.25 µm) and hydrogen (H_2_) as the carrier gas with a flow of 1.15 mL/min. The injection volume was 1 µL with a 50:1 split. The oven temperature was initially 80 °C, then increased to 200 °C with a gradient of 5 °C/min, and was held for 3 min.

The conversion of 3′-hydroxyacetophenone was monitored by chromatography with flame ionization detector (GC-FID, Agilent 7890B, Santa Clara, CA, USA) with CP-Chirasil-Dex CB capillary column (Agilent J&W, 25 m × 0.25 mm × 0.25 µm) and hydrogen (H_2_) as the carrier gas with a flow of 1.15 mL/min. The injection volume was 1 µL with a 100:1 split. The oven temperature was initially 90 °C, then increased to 150 °C with a gradient of 30 °C/min, and was held for 15 min. Then increased to 200 °C with a gradient of 30 °C/min, and was held for 2 min.

### 3.9. Storage Solution for Immobilized Biocatalyst

Storage solution contained (per litre): 0.1 M potassium phosphate buffer (pH 6.5), 4.7 M glycerol, and 2.0 × 10^−3^ M dithiothreitol (DTT).

## 4. Conclusions

Ketoreductase and glucose dehydrogenase were produced and co-immobilised via affinity interaction of His-tagged enzymes using affinity chromatography. Enzymes were applied in a set of biotransformation reactions in repeated batch flow-reactor mode. To date, this is the first immobilisation method used to immobilise these enzymes. The ratio of enzymes (crude extracts of ketoreductase and glucose dehydrogenase) was optimised (8:2 (*v*/*v*)). By performing co-immobilisation, the requirement of the cofactor was lowered, which is economically beneficial. Furthermore, applying the enzyme in this immobilised form significantly enhanced the tolerance toward higher substrate concentration. Immobilised system was also used for reduction of bulky ketones (4-phenyl-2-butanone and 3′-hydroxyacetophenone) and in comparison, with free enzymes can tolerate higher concentration of these substrates. Immobilised enzymes were applied in 20 and 13 repeated batch conversion cycles, respectively in the nonstop mode. During these cycles, enzymes retained 70% of the initial activity with a substrate concentration of 130 mM. Hence, this immobilisation technique has proven to be a quick, efficient, and a gentle method, and has the potential for scaling-up biotransformations.

## 5. Patents

Patent pending-Slovak patent PP50033–2020.

## Supplementary Materials

Figure S1: Presence of ketoreductase in *E.coli* cells, Figure S2: Different loading strategies for affinity immobilization, Figure S3: Increase in product concentration for various loading strategies, Figure S4: Comparison between use of crude cell extract and isolated enzymes, Figure S5: Activity of immobilized biocatalyst at different pH and temperature, Figure S6: Decrease in the initial enzymatic activity with increasing initial substrate concentration, Figure S7: Repeated biotransformations (1st–20th reaction)-130mM of substrate, Figure S8: Repeated biotransformations (1st–13th reaction)-190 mM of substrate, Figure S9: Decrease in activity of stored biocatalysts.
