# Supplementary material for: Semi-Continuous Flow Biocatalysis with Affinity Co-Immobilized Ketoreductase and Glucose Dehydrogenase
Source: Molecules. 2020 Sep 18;25(18):4278. doi: 10.3390/molecules25184278 (PMC7570937; doi:10.3390/molecules25184278)
Supplement: Supplementary file 1 [file molecules-25-04278-s001.pdf]

SUPPLEMENTARY MATERIAL

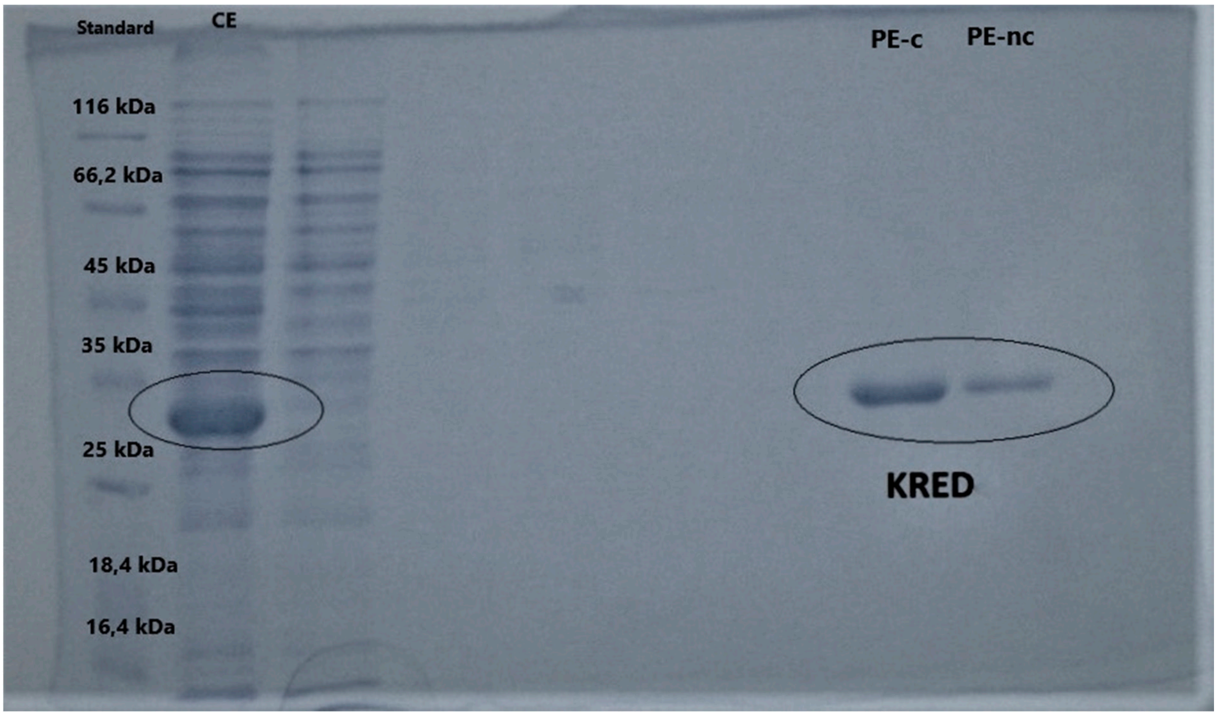

Figure S-1 Presence of ketoreductase in *E.coli* cells (**CE -**crude cell extract; **PE-c** – purified enzyme (concentrated); PE-nc -– purified enzyme (non-concentrated))

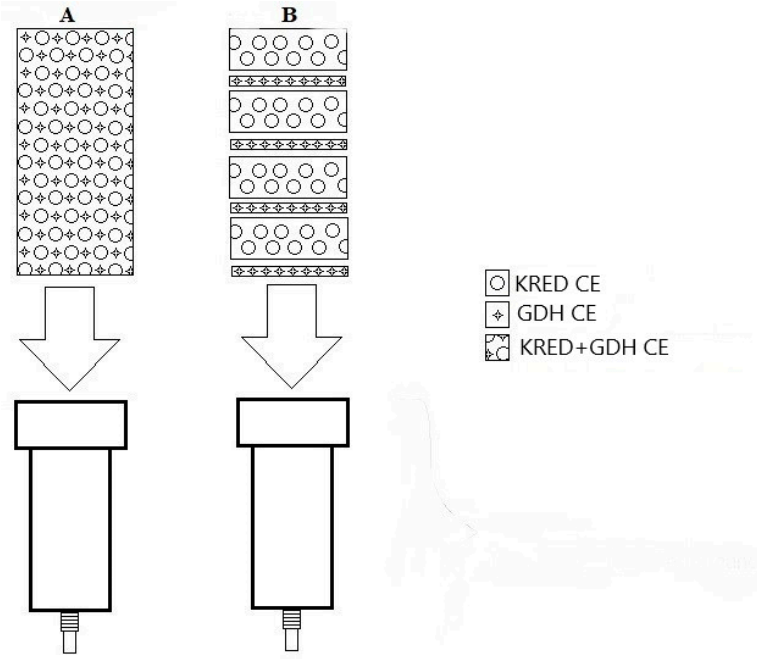

Figure S-2 Different loading strategies for affinity immobilization (**A**- °homogenous; **B**- zonal)

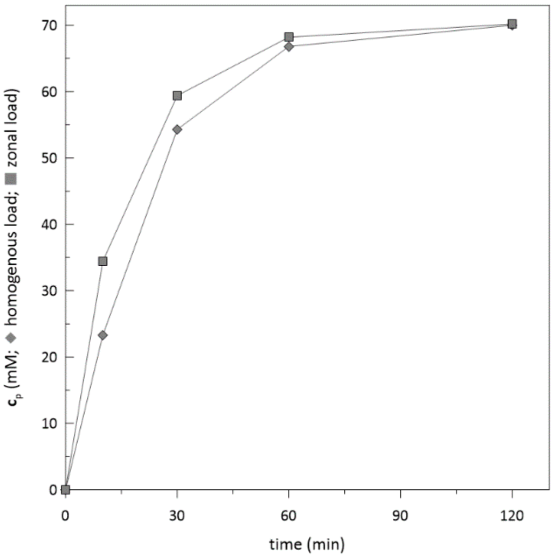

Figure S-3 Increase in product concentration for various loading techniques

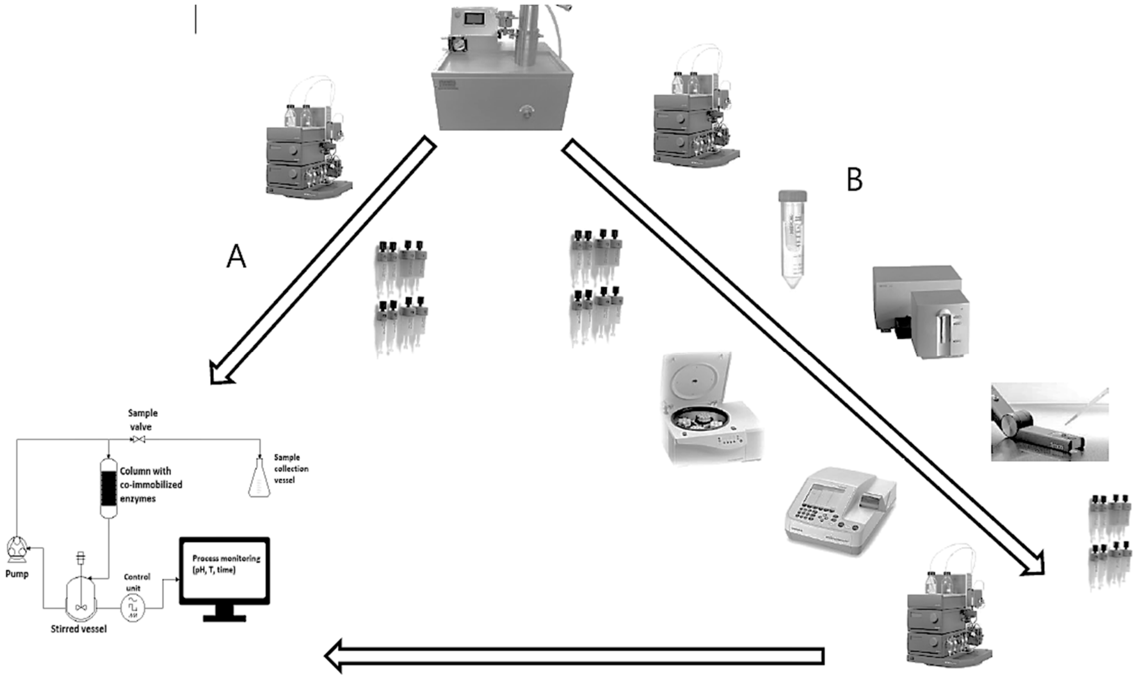

Figure S-4 Comparison between use of crude cell extract (route **A** combined crude cell extracts directed loaded onto column- reaction) and isolated enzymes (route **B** (for each enzyme)- isolation- concentration- desalinization- determination of protein presence and purity- activity assay-loading into the column-reaction) for immobilization into the column.

Time required for completion of route **A** ~ 1-2 hours and for route **B** ~ 5-6 hours

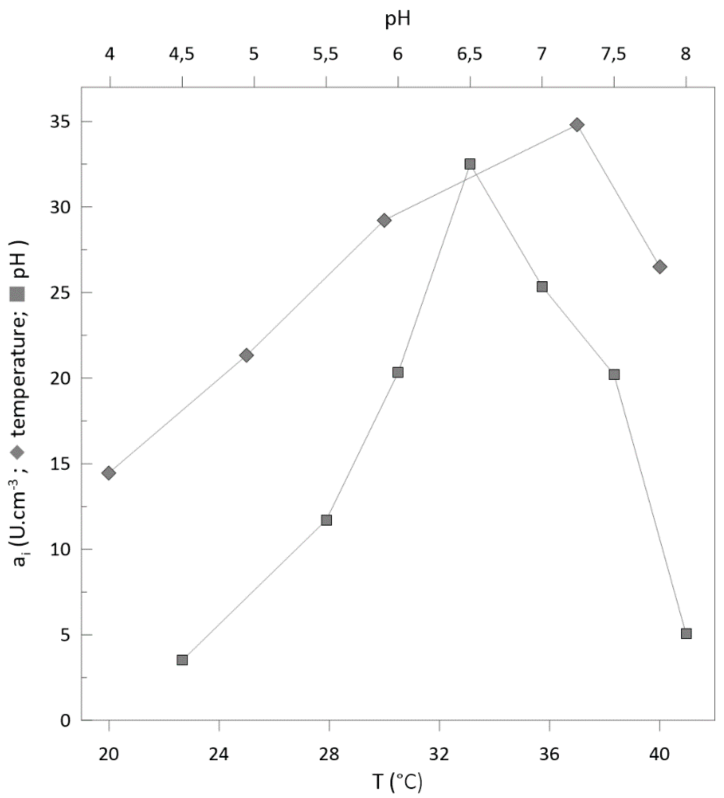

Figure S-5 Activity of immobilized biocatalyst at different pH and temperature

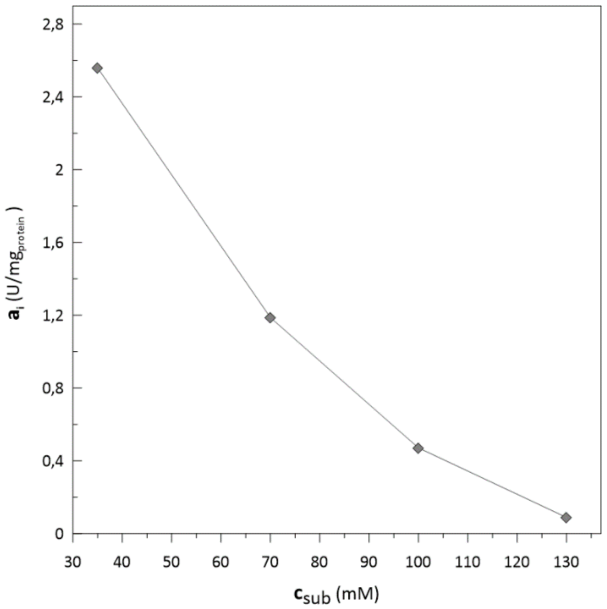

Figure S-6 Decrease in the initial enzymatic activity with increasing initial substrate concentration

Repeated biotransformations

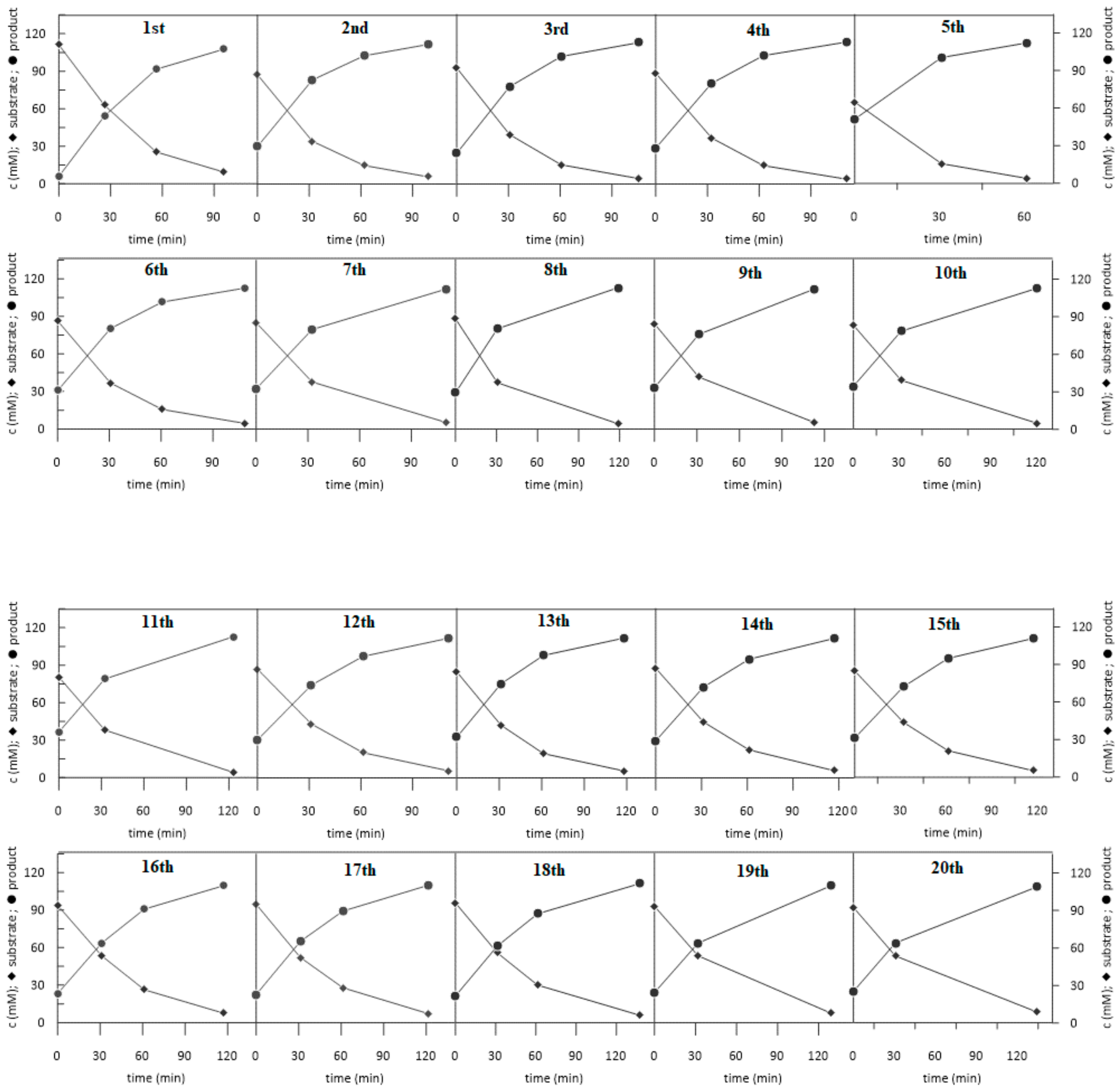

Figure S-7 Repeated biotransformations (1st - 20th reaction)-130mM of substrate

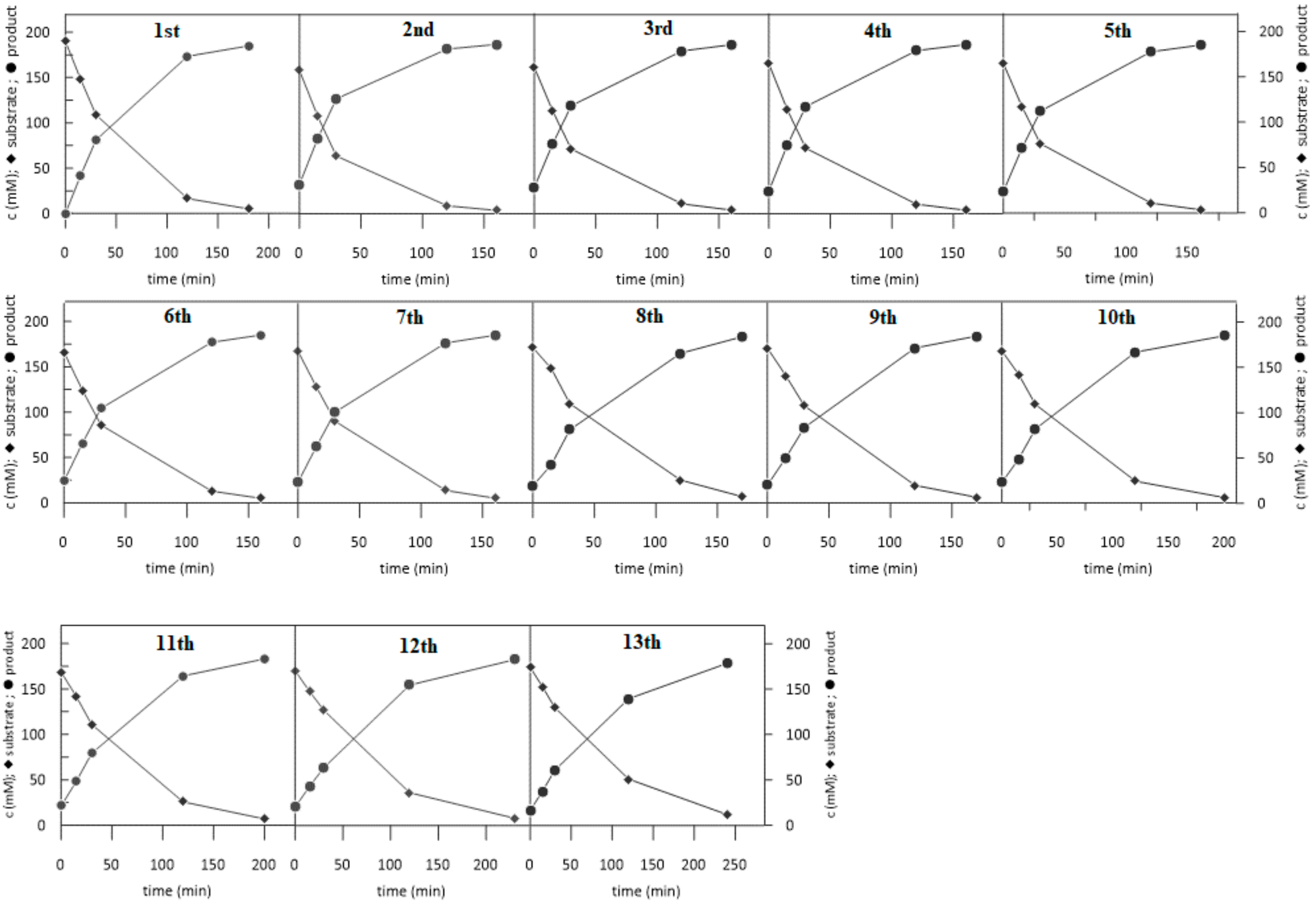

Figure S-8 Repeated biotransformations (1st - 13th reaction)-190mM of substrate

Storage of immobilized biocatalyst

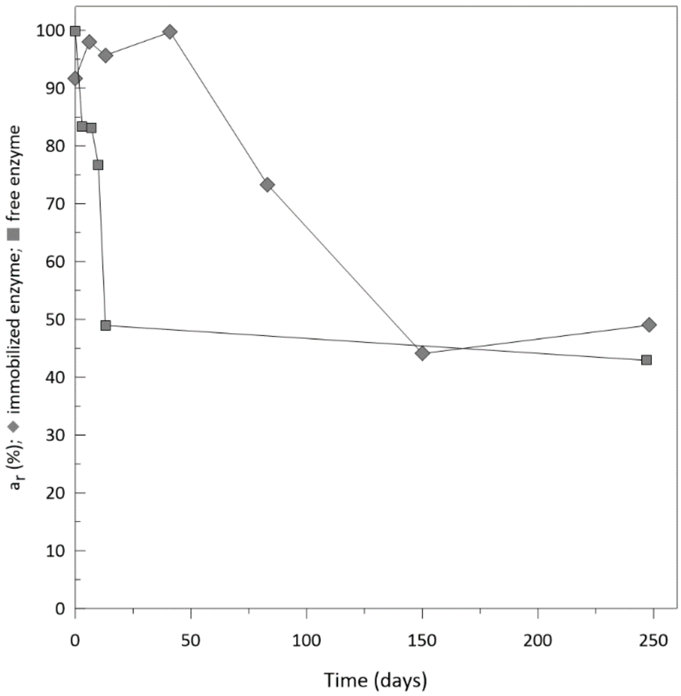

Figure S-9 Decrease in activity of stored biocatalysts
